# Kinetics and Kinematics of Shape Tracing in Children with Probable Developmental Coordination Disorder (pDCD)

**DOI:** 10.3390/children12010090

**Published:** 2025-01-15

**Authors:** Michal Hochhauser, Yfat Ben Refael, Esther Adi-Japha, Rachel Bartov

**Affiliations:** 1 Department of Occupational Therapy, Ariel University, Ariel 40700, Israel; yfatb@ariel.ac.il; 2 Faculty of Education, Bar-Ilan University, Ramat Gan 5290002, Israel; esther.adi-japha@biu.ac.il; 3 Faculty of Special Education, Orot Israel College, Elkana 4481400, Israel; rachel.b@orot.ac.il

**Keywords:** motor skills disorders, biomechanical phenomena, handwriting, cognition, visual–motor integration

## Abstract

Background: Children with developmental coordination disorder (DCD) exhibit visual–motor deficits affecting handwriting. Shape tracing, a key prerequisite for handwriting, supports motor and cognitive development but remains underexplored in research, particularly in objectively studying its role in children with DCD. Objectives: To compare the kinetics (pressure applied to the writing surface) and kinematics (spatial and temporal aspects) of shape tracing in children with pDCD to those of typically developing (TD) peers utilizing a digitized tablet. Methods: A total of 27 children with pDCD aged 7 to 12 years and 27 TD children matched by age and gender traced five unique shapes resembling print letters onto a digitized tablet. Participants’ performance measurements included precision, time, smoothness, velocity, and pressure. Results: The findings revealed lower precision, longer duration, more smoothness but less consistency, lower velocity, and less pressure application in the pDCD group. Conclusions: This research underlies the mechanisms of shape-tracing difficulties in children with DCD. Insights into early shape-tracing processes beyond product outcomes are essential for therapeutic and educational interventions, with digitized tablets offering a novel tool for assessing graphomotor skills in children with DCD.

## 1. Introduction

Developmental coordination disorder (DCD) is a neurodevelopmental disorder that affects motor skills development in children and is characterized by difficulties in movement acquisition and execution, which impacts their performance in activities of daily living [1]. Diagnostic criteria for DCD include motor abilities significantly below expectations for the individual’s age and learning opportunities, impacting daily activities and academic or professional achievements [2]. DCD affects about 5–6% of the population yet is underdiagnosed by healthcare and educational professionals [3].

Difficulties in motor performance include fine motor activities such as handwriting [4]. This fundamental skill is acquired during childhood and is necessary for all academic participation. Children with DCD often write slower with decreased overall legibility compared to typically developed children (TD) [5,6,7]. Bartov et al. [5] also examined fine motor handwriting aspects and found that children with DCD have weaker pen grasp, which may be associated with reduced legibility, form, fatigue, and prolonged writing duration. In the long term, this may lead to academic underachievement [8].

Handwriting is a complex skill. It is unique because it involves not just motor planning but also perceptual, linguistic, and executive processes [8]. Specifically, it constructs a delicate operation of the writing tool held by the palm and digits, is based on kinesthetic and tactile perception systems, also known as the haptic system, and is integrated with higher cognitive functions such as planning and monitoring [9]. Van Galens’ psychomotor theory [10] deconstructs the handwriting process, from conceptualizing ideas and sentence planning to writing, while managing size and shape, and making precise muscular adjustments for the smallest strokes. Moreover, visual feedback during writing plays an important role, as visual stimuli trigger real-time adjustments to motor planning schemes, creating a feed-forward mechanism that makes writing intuitive and automatic for TD children. Children with DCD, however, struggle in that area [11].

Children scribble first, then draw and copy shapes with increasing precision and control, and exercise what is known as graphomotor skills and visual–motor integration. These are well-known predictors of handwriting. For instance, seven-year-old children with handwriting difficulties showed visual–motor deficits on pencil–paper tasks as early as kindergarten [12]. Young children’s ability to copy geometric shapes is closely linked to their capacity to copy letters legibly [13], and handwriting legibility has been associated with cognitive planning skills [14]. With practice, finger movements typically become more fluid, letter shapes become steadier, and writing is less dependent on cognitive processes [15]. For example, Adi-Japha et al. [16] demonstrated in 5- to 8-year-old children that motor learning in a writing-like task (the “invented” letter task), reflected by improvements in velocity and legibility, predicted handwriting and reading performance one year later. In this task, children with DCD demonstrated learning rates comparable to their peers but with overall reduced accuracy and fluency [17]. A scoping review by Zwicker and Lee [18] compiled evidence supporting the importance of early intervention in young children, highlighting the critical need for early evaluation of graphomotor skills.

Standardized screening tools and tests in use, such as the Beery–Buktenica Developmental Test of Visual Motor Skill 6th edition (Beery VMI) [19] and the Fine Motor Integration subtest of the Bruininks–Oseretsky Test of Motor Proficiency 2nd Edition (BOT-2) [20], contain pen and pencil copying shapes tasks. Scores are recorded as dichotomous (either right or wrong) outcomes based on accuracy alone, without considering important variables that can be collected through technological advancements such as computerized assessments.

The availability and popularity of computer tablets offer computerized scoring of handwriting readiness, allowing for the evaluation of the process rather than just the outcome. Recent studies focused on early intervention and have developed tablet-based assessments with different methodologies. Chu and Krishnan [21] analyzed different accuracy parameters, such as alignment and roundness, in a shape-copying task for children aged 5–10. Thorsson et al. [22] administered a gamified tracing task to 10-year-old children to assess motor coordination by collecting directional and spatial measures. Both studies compared their results to the Beery VMI and yielded initial results that identified children with visual–motor difficulties.

Furthermore, while many studies have shown that children with DCD perform poorly in graphomotor skills, such as shape *copying*, research on impaired shape *tracing* in children with motor difficulties is limited. Schott et al. [23] found that children with DCD were slower in a trail-tracing test than their peers. Zwicker et al. [24] conducted a study with children aged 8–12, with DCD and their typically developing peers TD, who practiced a trail-tracing task while their brain activity was measured. The study revealed that children with DCD showed poorer tracing accuracy, suggesting that, compared to TD peers, they demonstrate under-activation in cerebellar–parietal and cerebellar–prefrontal networks and in brain regions associated with visual spatial learning. An additional study found that 7–12-year-old school children with DCD were significantly worse at a 3D tracing task than the age-matched typically developing children and is associated with 2D drawing [25,26].

However, while these studies measured speed and accuracy, significant indicators of the motor and cognitive processes identifying children with DCD in tracing tasks, such as velocity (i.e., measure of the pen or finger movement as it accounts for diagonal motion, not just motion along a single axis) [27,28], pressure and smoothness have not yet been investigated. Proficient handwriting refers to the ability to produce legible text in a reasonable amount of time and is related to temporal and spatial consistency across repetition over time due to the minimized physical cost of movements (e.g., less jerky movements) [6,29]. Faster velocity indicates how quickly a person can produce strokes or letters, which often reflects greater handwriting fluency and motor efficiency, while slower velocity may indicate motor difficulties or cognitive overload [30]. Smoothness, measured by Rényi entropy, captures how smooth writing movements are using acceleration, providing insight into control and stability. A lower rate of change in acceleration indicates, on the one hand, smoother, more predictable movements [31], but may reflect, on the other hand, reduced consistency in task performance of shape tracing, highlighting challenges in visual–motor coordination.

Kinematic and kinetic assessments in shape tracing can provide insights into how children with DCD process and execute movements that require precision, coordination, and fluidity, shedding light on the mechanisms underlying their motor execution difficulties in graphomotor tasks. These insights can significantly enhance our understanding of motor control and learning in children with DCD, offering practical applications in both clinical and educational settings. By highlighting the relationship between kinetic and kinematic measures and motor learning, this study could lead to more targeted assessment methods and interventions. Additionally, incorporating digitized tools and real-time feedback mechanisms could transform current approaches to early detection and support for graphomotor challenges, ultimately improving outcomes for children with DCD.

Thus, we hypothesized that the kinetics and kinematics involved in performing a shape-tracing task on a tablet would be inferior in children with DCD compared to TD children. By doing so, we harvested the technological abilities of affordable technology to enrich the knowledge about evaluative tasks of pre-writing skills of children with and without DCD.

## 2. Materials and Methods

### 2.1. Participants

A total of 58 children aged 8 to 12 years, 34 (59%) boys and 24 (41%) girls, participated in the study. Data of 27 children with DCD or high probability of DCD (pDCD) were derived from a former study [5]. They matched the criterion through standardized tests and were verified with school counselors and class teachers. They were matched for age (up to a 6-month difference) and gender with the control group (TD) (*n* = 31). The control group was formed through a word-to-mouth approach. It consisted of typical children based on parent reports and without probability of DCD based on the manual dexterity subtest of MABC-2. Table 1 shows the sample characteristics.

The study’s exclusion criteria (both groups) were children with emotional disorders, autism, physical limitations, visual or hearing impairments, neurological disorders, or special education classes. All children in the study were Hebrew speakers.

### 2.2. Research Tools

Movement Assessment Battery for Children. The MABC-2 [32] is a standardized, valid test to identify children with DCD or pDCD between 3 and 16 years old. We used the appropriate battery for ages 7.00 to 10.11 years. Each battery includes eight motor tasks divided into three subdomains: manual dexterity, ball skills, and balance. Scores between 6% and 15% indicate at risk for motor impairment; scores lower than 5% indicate the presence of a motor disorder.

Tablet Shape-Tracing task. We used a Wacom Cintiq version 13-HD (Cintiq 13 HD Graphic Pen Tablet for Drawing) computerized tablet as a digital writing surface. It was covered with a screen protector to increase friction, similar to paper. The tablet was positioned about 2 cm from the table’s edge, and the children drew with a stylus similar to a regular pen. The tracing task consisted of five abstract shapes specifically designed for the study, including straight and curved lines resembling Hebrew print and script letters (also similar to English letters, which children learn at a later stage), presented in a randomized order to minimize sequence effects. Participants wrote on a lined sheet, receiving visual feedback on their products while the tablet recorded the written traces. The position of the pen tip was registered with a spatial resolution of 0.5 mm at a sampling rate of 133 Hz with an accuracy of 0.1 mm. Measurements included precision (i.e., the offset between the original shape line and the traced line measured by dynamic time warping, DTW) [33], time (i.e., duration of writing the letter), mean velocity, smoothness, and pressure. Smoothness was computed using the velocity time-series data derived from the positional information recorded by the tablet. The tablet captured the instantaneous velocity of the pen tip, which was calculated as the rate of change in position over time. The variability and regularity of this velocity were analyzed by constructing a probability distribution of the velocity values. To quantify smoothness, we applied Rényi entropy, which measures the complexity or predictability of the velocity distribution [34]. Lower Rényi entropy values indicated more predictable and smooth movements, while higher values reflected less regular and jerkier movements. Additionally, we measured velocity (Z), which represents the instantaneous movement speed [35]. While Rényi entropy focuses on the diversity and complexity of velocity patterns to assess variability or regularity, velocity (Z) captures real-time movement dynamics, providing complementary insights into handwriting behavior.

Calibration was based on average pressure levels among children with TD, as determined in a previous study [5], which provided the conversion of pressure levels in the Wacom tablet to Newtons.

Production time was computed based on the digitized data, from the first touch of the pen tip on the tablet until task completion. The time it took the participant to produce the shape was divided into on-tablet time (i.e., the total time the pen tip touched the tablet) and off-tablet time (i.e., the total time the stylus did not touch the writing surface). The digitized tablet provided a flag measure of the on-tablet/off-tablet contact calibrated by the axial pressure of the writing stylus on the tablet surface. The on-tablet time was measured directly. The off-tablet time was computed as the difference between the overall production and on-tablet times. These two components were summed to calculate the overall production time, equivalent to the “duration” value reported in the results. By differentiating on-tablet and off-tablet times, we ensured precise tracking of the participants’ writing behavior and task completion.

### 2.3. Procedure

The research was approved by the (blinded) University Ethics Review Board (blinded number). Parents of both groups signed informed consent. Children classified as the pDCD group met the MABC-2 and the DCDQ criteria. Both groups were administered the manual dexterity segment of the MACB-2 and the tablet shape-tracing task individually in a quiet room at an appropriately sized table by a trained occupational therapist. Following a trial trace, using non-experimental shapes to familiarize themselves with the tablet interface and task requirements, each participant traced five shapes sequentially as they appeared on the screen. During the task, participants were instructed to prioritize accuracy over speed and trace the shapes as closely as possible to the provided outline. The procedure took about 20 min per child.

Data processing was conducted using MATLAB^®^ version R2023a, and statistical analyses were performed using IBM SPSS (Version 27). We utilized repeated measure analysis of variance (ANOVAs) to evaluate group differences across the shape-tracing metrics, with a significance threshold set at *p* < 0.05. Additionally, post hoc comparisons were conducted using Bonferroni adjustments. At the start of the research, we predefined that up to 5% of trials with incomplete or highly deviant performances (e.g., >3 SD from the group mean) would be excluded to maintain data quality. However, due to strong participant adherence and minimal technical issues, only 0.004% of trials were excluded, and 0.006% were winsorized to account for outliers. These procedures did not alter the findings, which remained consistent upon reanalysis.

## 3. Results

The performance of the two groups—TD children and children with pDCD—was compared across various measures of shape tracing: shape precision, time, mean velocity, smoothness, and pressure. Performance Overview: on average, TD children outperformed children with DCD across all shape measurements (Table 2).

### 3.1. Shape Precision

**Figure 1:**
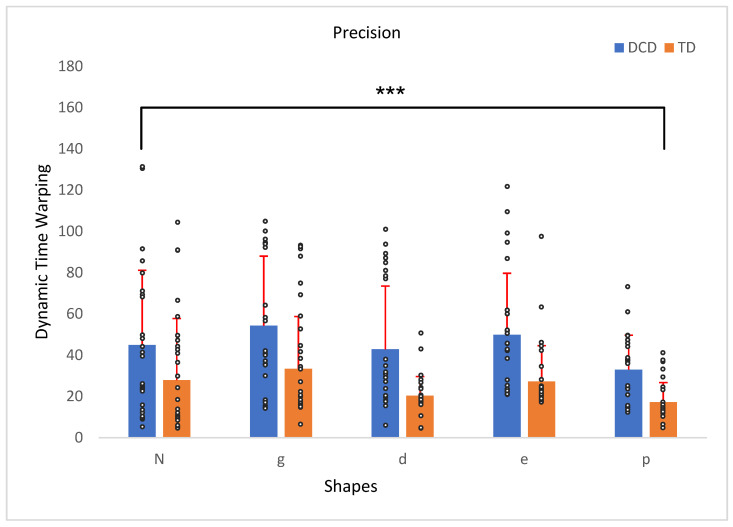
Precision/offset of the traced shape in relation to the original shape (mm) of the TD children compared to the children with DCD. Error bars represent the standard error (SE), calculated using the standard deviation (SD) and adjusted for the sample size. *** *p* < 0.001.

There was a significant main effect for precision, *F*(4, 208) = 0.64, *p* < 0.001, η^2^ = 0.11, indicating a moderate effect size. This main effect indicated that shape precision varied by letter traced. Specifically, the precision for shape “*p*” showed the most substantial difference, particularly when compared to shapes “g” and “e” (*p* < 0.001 and *p* = 0.03, respectively). A significant between-subjects effect for group, *F*(1, 52) = 17.16, *p* < 0.001, η^2^ = 0.24, confirmed that precision differed significantly between the TD and DCD groups. However, no significant interaction was found between precision and group, *F*(4, 208) = 0.32, *p* = 0.87 (Figure 1).

### 3.2. Duration of Tracing

**Figure 2:**
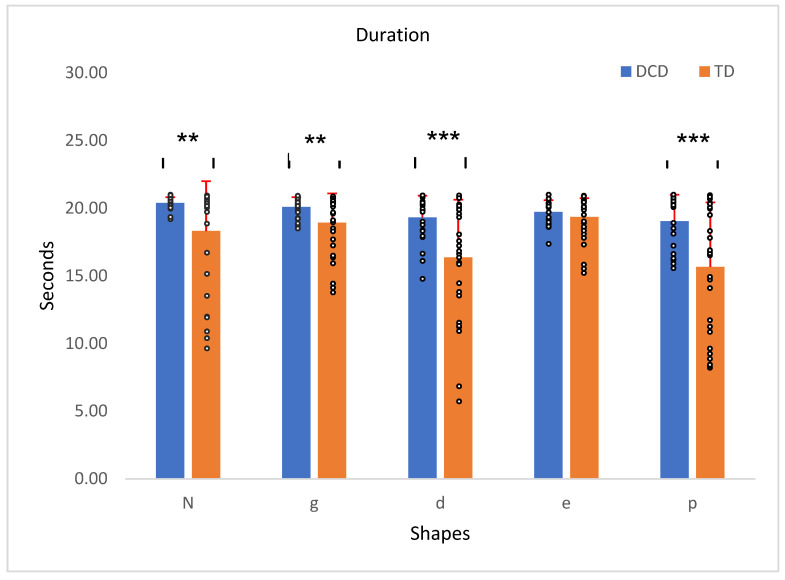
Duration of the TD children’s traced shape compared to those with DCD (second). Error bars represent the standard error (SE), calculated using the standard deviation (SD) and adjusted for the sample size. ** *p* < 0.01, *** *p* < 0.001.

A between-group effect indicated that children with DCD took significantly longer to complete the tracing task across all shapes, *F*(1, 56) = 16.65, *p* < 0.001, η^2^ = 0.23. A significant main effect for duration was observed, *F*(4, 224) = 13.05, *p* < 0.001, η^2^ = 0.19, suggesting that the duration varied across shapes. Additionally, a significant interaction between duration and group, *F*(4, 224) = 2.45, *p* = 0.05, η^2^ = 0.42, indicated that the group differences depended specifically on individual shapes. Independent *t*-tests revealed a significant difference in duration between the DCD and TD groups in four out of five shapes (Figure 2).

### 3.3. Smoothness (vs. Jerkiness)

**Figure 3:**
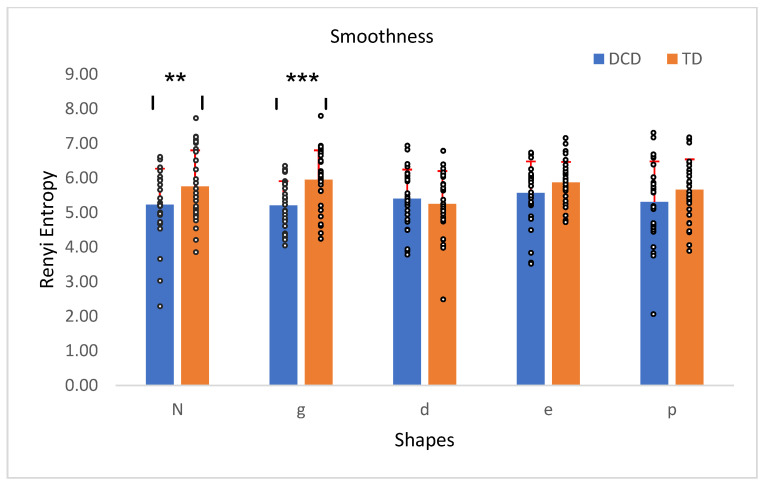
The smoothness of the TD children’s traced shape compared to the children with DCD (Rényi entropy). Error bars represent the standard error (SE), calculated using the standard deviation (SD) and adjusted for the sample size. ** *p* < 0.01, *** *p* < 0.001.

No significant main effect was found, *F*(4, 224) = 2.24, *p* = 0.07, suggesting no notable differences in smoothness across shapes. However, a significant between-group effect was found, *F*(1, 56) = 4.67, *p* = 0.04, η^2^ = 0.08, indicating smoother movements in DCD children than in TD. A significant interaction was also found between group and smoothness, *F*(4, 224) = 2.94, *p* = 0.02, η^2^ = 0.05, indicating a moderate effect size, and suggesting smoothness varied differently between the TD and DCD groups in specific shapes. Independent *t*-tests revealed a significant difference in smoothness between the DCD and TD groups in two out of five shapes (Figure 3).

### 3.4. Velocity

**Figure 4:**
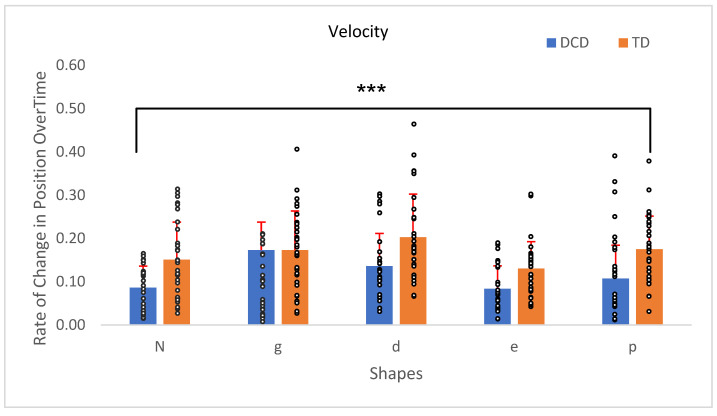
Velocity of the TD children’s hand movement compared to the children with DCD. Error bars represent the standard error (SE), calculated using the standard deviation (SD) and adjusted for the sample size. *** *p* < 0.001.

A between-group effect, *F*(1, 54) = 15.86, *p* = 0.001, η^2^ = 0.18, confirmed that overall, TD children exhibited greater velocities than children with DCD. A significant main effect of velocity was found, *F*(4, 216) = 12.42, *p* < 0.001, η^2^ = 0.19, indicating that velocity varied significantly across different shapes. However, no significant interaction effect was observed between velocity and group, *F*(4, 216) = 0.91, *p* = 0.46 (Figure 4).

### 3.5. Pressure

No significant main effect of pressure was found, *F*(4, 224) = 1.07, *p* = 0.37, nor was there a significant interaction between group and pressure, *F*(4, 224) = 1.47, *p* = 0.21. However, a significant between-group effect was found, *F*(1, 56) = 10.83, *p* = 0.002, η^2^ = 0.16, indicating that TD children applied more pressure than children with DCD (Table 1).

In summary, the analysis revealed significant differences between typically developing children and children with DCD across various measures of shape-tracing performance. The TD group consistently performed better in precision, speed, and movement velocity. In contrast, the DCD group took longer and exhibited more movement variability and smoothness—but less consistency. As indicated by partial eta-squared values, the effect sizes ranged from small to moderate, suggesting meaningful differences between the two groups in several performance measures.

## 4. Discussion

This study investigated the kinetics and kinematics of shape tracing in children with DCD compared to their typically developing (TD) peers. The findings revealed significant differences between the two groups in precision, time, velocity, smoothness, and pressure.

Children with DCD demonstrated lower precision, required more time to complete shape-tracing tasks, and had more pauses (i.e., segmentation; off-tablet’) than their TD counterparts. This aligns with the understanding that DCD affects the execution of controlled motor tasks [7]. The prolonged duration and reduced precision suggest difficulties in motor planning and execution, as well as challenges in adapting motor responses through the automatization of feedback, as reported in the literature [36]. These observations align with the literature indicating that children with DCD struggle with integrating the motor and cognitive processes necessary for fluid and accurate writing [6,9,11] and the fragmented and less efficient strategies these children use for motor planning and execution [37]. Additionally, the segmented performance may indicate impaired executive functioning, which impacts the smooth execution of learned motor tasks and contributes to the observed challenges in forming cohesive and accurate representations [38]. Moreover, previous studies have highlighted the reliance of children with DCD on compensatory strategies, such as increased pauses and dependence on visual feedback, to perform motor tasks [36]. While these strategies can help mitigate some challenges, they often result in fragmented and inefficient motor execution, as observed in our participants. These observations should prompt further investigation of the role of visual feedback in motor learning and its implications for developing graphomotor skills in children with DCD. Evidence from Tseng et al. [39] demonstrated that children with DCD exhibit proprioceptive deficits, particularly in wrist position. The study found that children with DCD showed increased joint position variability during active wrist matching and higher thresholds for passive displacement compared to TD peers. Furthermore, these proprioceptive deficits correlated significantly with manual dexterity and balance, supporting the notion that proprioceptive dysfunction in the wrist–hand complex contributes to fine motor challenges in DCD.

In the current study, the TD group demonstrated shorter durations for each letter. Surprisingly, the TD group presented greater variance, though developmental disorders such as DCD are often associated with greater variance in performance. This finding may reflect the heterogeneity of motor proficiency within the TD group, as it included children with MABC-2 scores ranging from just below the 25th percentile (not at risk for DCD but lower in motor performance) to much higher functional percentiles. These results suggest categorizing participants into narrower subgroups based on functional levels could provide clearer insights. Since functional heterogeneity is the norm in real-world contexts, future research can consider stratifying groups using additional functional or behavioral metrics (e.g., dividing TD children into higher and lower motor proficiency levels).

It is also important to note that while one might initially expect children with TD to exhibit *lower* Rényi entropy values due to their smoother handwriting, the results revealed *higher* entropy values for this group. This counterintuitive finding may be explained by their greater precision and ability to correct their writing. Specifically, children with TD demonstrated a propensity to realign their writing trajectory with the target line, resulting in movements that introduced dynamic irregularities. This behavior was particularly pronounced in letters with angles or crossed lines, for example, 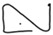 or 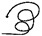, which require precise and corrective actions. Thus, the higher entropy values reflect *consistency* introduced by their corrective precision rather than a lack of smoothness.

Kinematic analysis of shape tracing reveals notable within-difference variations in how different shapes are processed. These differences often relate to each shape’s complexity and inherent properties, such as the number of corners, curvature, and symmetry [40,41]. For example, simpler shapes like circles and squares tend to exhibit smoother and more consistent tracing velocities, whereas more intricate shapes with multiple angles or irregular curves may show greater variability in speed and accuracy [42]. This variability can be attributed to the need for frequent adjustments in motor control, as the tracing hand must slow down at corners or change direction rapidly to maintain accuracy [4]. Such adjustments can impact the overall fluency of the movement, leading to a less uniform kinematic profile across different shapes [43].

Moreover, differences in kinematic measures such as acceleration, deceleration, and pauses within the tracing process can reflect how children with DCD adapt their motor planning and control to the shape’s unique requirements [44]. Shapes with complex structures may necessitate greater cognitive and motor resources, resulting in increased pauses or changes in velocity as the individual recalibrates their hand movements [45]. This can indicate the children’s reliance on visual feedback to correct and adjust their motor actions during more difficult tracing tasks [5]. The within-differences in tracing kinematics underscore the importance of considering shape characteristics in assessing motor skills, as they provide insights into the adaptability and precision of motor control mechanisms in children with and without motor coordination challenges [46].

The findings also highlighted that TD children displayed greater velocity; more efficient and fluent handwriting was characterized by faster execution of strokes. This may reflect reduced hesitation during writing, suggesting that TD children are more confident in their movements and require fewer pauses or corrections. This efficiency, combined with their ability to execute corrective movements, when necessary, underscores the complexity of their handwriting dynamics. While faster strokes are typically associated with smooth movements, the interplay between speed, precision, and corrections in the TD group may introduce subtle irregularities, contributing to higher entropy values. The decreased velocity in children with DCD can be linked to an impaired feed-forward mechanism, which hinders their ability to plan and execute motor actions efficiently and adaptively based on visual feedback [11]. The reliance on visual feedback is critical for motor learning and performance, as emphasized by [18], who noted its particular importance in children with DCD [5]. The observed differences in velocity and precision reflect the challenges faced by children with DCD in coordinating motor actions and developing automated motor skills. This inability to produce fluid, consistent motor outputs indicates fundamental difficulties in motor planning and execution, which aligns with Wilson et al.’s [47] findings on the limitations in motor coordination and the reliance on less efficient, compensatory motor strategies in this population. This is also supported by the work of Piek and Skinner [48], which highlights deficits in motor coordination and timing in children with DCD, suggesting that their motor actions lack fluidity due to impaired sensorimotor integration. However, while these findings align with the previous literature, they raise questions about the role of task complexity in motor planning difficulties. Missiuna et al. [49] emphasized that as task demands increase, children with DCD exhibit even greater motor inconsistencies, pointing to a potential threshold effect where task complexity exacerbates coordination challenges. This highlights the importance of investigating how varying graphomotor task difficulties influence motor control strategies in this population.

Additional findings show that children with DCD applied less pressure on the writing surface than TD children. This finding is inconsistent with the authors’ previous study [50], where the findings revealed typical handwriting writing pressure in the same group of children with DCD. This discrepancy may reflect their ability to rely on practiced, automatic motor skills and compensatory strategies during handwriting. In contrast, the reduced pressure during tracing highlights their struggles with less familiar tasks requiring real-time adjustments, greater visual feedback, and greater dynamic motor control. The interplay between visual–motor integration and less haptic feedback in the tracing task likely contributes to the observed disparities, emphasizing the need for targeted interventions that enhance proprioceptive and visual feedback systems to support motor learning in early graphomotor skills.

Theoretical implications of these findings extend to models of motor control and learning. The reliance on feedback-based adjustments observed in children with DCD challenges the adequacy of feed-forward control models to fully explain their motor execution patterns, specifically in shape precision in a tracing task. Notwithstanding, our findings suggest an increased dependency on sensory input to guide movement, particularly in tasks requiring complex visual–motor integration. Moreover, the variability in motor execution raises questions about task complexity thresholds, supporting the idea that heightened task demands exacerbate motor challenges. Future research could explore how interventions targeting feedback mechanisms and proprioceptive training may enhance motor control and reduce reliance on compensatory strategies.

This study has several limitations. Firstly, the relatively small sample size may limit the generalizability of the findings to broader populations of children with DCD. Additionally, while the digitized tools used in this study provided detailed insights into kinetic and kinematic aspects of tracing, they may not fully replicate real-world conditions of clinical handwriting assessment tools. Lastly, future research could benefit from a longitudinal study investigating the association between shape-tracing aspects and handwriting to provide a more comprehensive understanding of assessment outcomes and graphomotor challenges.

## 5. Conclusions

This study’s results align with previous findings on perceptual motor coordination difficulties in children with DCD, highlighting the need for further research on multi-sensory training and visual–haptic integration as potential areas of exploration. The real-time analysis of shape-tracing kinetics and kinematics revealed the challenges children with DCD face in fine motor control and visual–motor integration compared to their peers. These group differences in tracing precision, timing, movement velocity, and consistency underscore the complexity of motor learning in this population and the value of structured assessment practices to understand and address these difficulties.

The findings emphasize the importance of digitized tools, such as tablets, for real-time objective assessments that enable early detection of graphomotor skill challenges in children with DCD. These tools allow clinicians and educators to gain valuable insights into shape-tracing processes and motor control mechanisms, supporting evidence-based assessment practices. Integrating visual–haptic feedback into these assessments may offer a clearer understanding of motor learning processes, fostering cohesive movements and improving task efficiency.

Overall, the results underscore the importance of multifaceted approaches that combine early assessments, tailored support strategies, and adaptive learning methods to help children with DCD develop foundational motor and handwriting skills. Further research is needed to determine how these insights can inform interventions in educational and therapeutic contexts.

## Figures and Tables

**Table 1:** Study sample characteristics, *N* = 58.

Variable	pDCD Group *N* = 27		TD Group *N* = 31		Ӽ^2^
	*n*	%	*n*	%	
Gender					0.6
Boys	17	63	17	55	
Girls	10	37	14	45	
	*M*	*SD*	*M*	*SD*	*t*(56), *p*
Age (year)	9.64	1.03	9.97	1.18	1.14, 0.26
	pDCD Group *N* = 27		TD Group *N* = 31		
MABC-2	*M*	*SD*	*M*	*SD*	*t*(56), *p*
Manual dexterity	14.19	3.3	55.48	27.79	9.68, <0.001

Note. pDCD = probability for developmental coordination disorder; TD = typical development.

**Table 2:** Tracing task performance; between-group differences, *N* = 58.

Variable	pDCD Group *n* = 27		TD Group *n* = 31					
	Mean	SD	Mean	SD	*df*	*F*	*p*	*η* ^2^
Precision ^1^	45.9	21.78	28.2	15.53	1, 56	12.96	0.001	0.19
Duration (sec) ^2^	19.5	1.09	17.43	2.94	1, 56	11.78	0.001	0.17
Smoothness ^3^	5.34	0.61	5.7	0.63	1, 56	4.67	0.04	0.08
Velocity ^4^	0.11	0.07	0.17	0.07	1, 56	9.23	0.004	0.14
Pressure ^5^	0.63	0.09	0.69	0.1	1, 56	5.46	0.02	0.09

Notes. ^1^ Precision variable is calculated as DTW. ^2^ Duration variable relates to seconds. ^3^ Smoothness variable relates to Renyi entropy calculation. ^4^ Velocity is calculated as the rate of change in position over time (e.g., horizontal and vertical axes). ^5^ Arbitrary unit; for conversion to Newton, see https://www.mdpi.com/article/10.3390/children10091534/s1, accessed on 1 January 2024 [5].

## Data Availability

The data presented in this study are available at the request of the corresponding author due to ethical reasons.
